# Clinicopathological Study of Mucormycosis in COVID-19 Patients: Experience From a Tertiary Care Center in South India

**DOI:** 10.7759/cureus.23016

**Published:** 2022-03-10

**Authors:** Sangeetha Kandasamy, Srinivas Muthuraju, Arumugam Vasugi, Megala Chandrasekar, Roopmala Murugan, Poovizhi Inbasekaran, Prabu R

**Affiliations:** 1 Pathology, Vinayaka Missions Kirupananda Variyar Medical College & Hospitals, Salem, IND; 2 Neurology, Thirunelveli Medical College and Hospital, Thirunelveli, IND; 3 Pathology, Sri Ramachandra Institute of Higher Education and Research (SRIHER), Chennai, IND; 4 General Internal Medicine, Vinayaka Missions Kirupananda Variyar Medical College & Hospitals, Salem, IND

**Keywords:** fungal stain, uncontrolled diabetes, steroids, polymerase chain reaction, cytokine storm, immuno suppresion, covid-19, mucormycosis

## Abstract

Background: The coronavirus disease 2019 (COVID-19) pandemic has taken the world to different dimensions. With the surge of the second wave in India, the number of cases with mucormycosis has increased. Mucormycosis is a potentially life-threatening, opportunistic, invasive, fungal infection that can occur in immunocompromised states. The aim of the study is to analyze the clinicopathological parameters of patients with mucormycosis in the surge of the second wave of COVID-19.

Materials and Methods: All cases of mucormycosis reported in the Department of Pathology in a tertiary care centre in South India from March 2021 to June 2021 were included in the study. The patient details were retrieved from the Medical Records section. The patient characteristics, location, associated comorbidities, management and treatment outcomes were analyzed and compared to similar studies reported worldwide.

Results: Of 58 cases, 38 (65%) were males and 20 (35%) were females. The ages ranged from 34 to 77 years. Severe acute respiratory syndrome coronavirus 2 (SARS-CoV-2) was detected in 46 patients in reverse transcription polymerase chain reaction (RT-PCR) with high-resolution computed tomography (HRCT) Chest changes noted in 54 patients. Associated comorbidities were noted in 52 patients, with uncontrolled diabetes mellitus (46 patients; 88%) being the most common. Location was commonly in nasal and paranasal sinuses (43%), followed by orbital (2%), cerebral (10%) and pulmonary (8%) areas. Among the paranasal sinuses, the maxillary sinus was commonly involved. Mixed fungal infections (Aspergillus sp. and Candida sp.) were noted in eight (14%) cases. Oxygen therapy was given in 85% of cases; 30% of cases needed ventilator support; corticosteroid therapy was initiated in 49 patients, tocilizumab in six patients as treatment for mucormycosis. Amphotericin B was administered in 59% of patients based on clinical findings alone. After histopathological confirmation, 90% of them received amphotericin. Functional endoscopic sinus surgery (FESS) was done in 96% of cases, among them 45% underwent extensive surgical debridement and 15% underwent orbital decompression. Orbital exenteration (2%) was the other modality of management.

Conclusion: Detailed analysis of clinicopathological features suggests the possibilities of immunosuppression (due to diabetes and use of corticosteroids in treatment of COVID-19) and COVID-19 (endothelial damage, cytokine storm) being the pathogenesis associated with the sudden surge of mucormycosis.

## Introduction

The coronavirus disease 2019 (COVID-19) caused by the novel SARS-CoV-2 has been associated with a myriad of disease patterns, ranging from mild cough to life-threatening pneumonia [1]. The daily detected cases of COVID-19 in India were low till mid-February 2021 after which there was a sharp rise indicating the surge of a second wave. Studies identified double mutant and triple mutant strains of SARS-CoV-2 which are considered to be more pathogenic than the initial strains, with SARS-CoV-2 (B.1.617 lineage) being the highly infectious double mutant variant [2].

A new threat in the second wave was the sudden increase in the number of mucormycosis, which is a potentially life-threatening, opportunistic, invasive, fungal infection commonly called the “Black fungus”. By June 7, 2021, about 28,252 cases of mucormycosis had been recorded by the Indian Health Ministry. A complex spectrum of factors was considered to be facilitating the rise in mucormycosis. The high oxygen saturation in COVID-19 is an ideal environment for the germination of sporangiospores. Another potential cause is immunosuppression caused by systemic immune alterations by COVID-19, coupled with uncontrolled diabetes mellitus (DM), poor glycemic control, steroid therapy, pre-existing paranasal and airway diseases such as asthma, chronic obstructive pulmonary disease (COPD) and other associated comorbidities. The third factor is prolonged hospitalization and oxygen need with or without ventilator support and also possible nosocomial sources [3]. Furthermore, the overexpression of cytokines and inflammatory markers such as interleukins (IL-1, IL-6, IL-8, IL-120 and IL-12), CXCL-10, tumour necrosis factor-α (TNF-α), IFN-λ, IFN-β, macrophage inflammatory protein-1α (MIP-1α) and monocyte chemoattractant protein-1 (MCP-1) called the “cytokine storm”, and decreased T-helper (CD4+ and CD8+) cells makes the patient/individual vulnerable to a wide range of opportunistic bacterial and fungal co-infections [4].

Baker in 1957 [5] coined the term “mucormycosis” which was previously described as “phycomycosis or zygomycosis” by Paltauf in 1885 [6]. Mucormycosis is a disease caused by various fungi. Rhizopus is one of the commonest fungi causing this disease. The mode of transmission is through inhalation of spores. Surgical debridement and amphotericin-B remain the mainstay of treatment. The angio-invasive nature and rapidity of dissemination of these molds make it potentially life-threatening. Early diagnosis and treatment may prevent the rapid progression of the disease since the reported mortality rates from intra-orbital and intracranial complications are 50-80%. The fatality rate is as high as 90% in cases of intracranial involvement [7]. The present study aims to obtain a detailed clinic-pathological analysis of COVID-19 patients with mucormycosis, associated comorbidities, therapy administered and patient outcomes.

## Materials and methods

This is a retrospective study that included all cases of mucormycosis reported in the Department of Pathology, in a tertiary care centre in South India from March 2021 through June 2021. The patient details were retrieved from the Medical Records section. The patient characteristics, location, radioimaging studies, associated comorbidities, management and treatment outcomes were obtained, recorded and analyzed. The observation findings were compared to similar studies reported worldwide. A literature search was conducted in the electronic databases of Pubmed and Scopus articles using the keywords “COVID 19 and Mucor”, “SARS-CoV-2 and Mucormycosis”, “Mucorales”, “Rhizopus”, and “Mucormycosis” to look for similar case series and the findings were compared.

## Results

**Figure 1:**
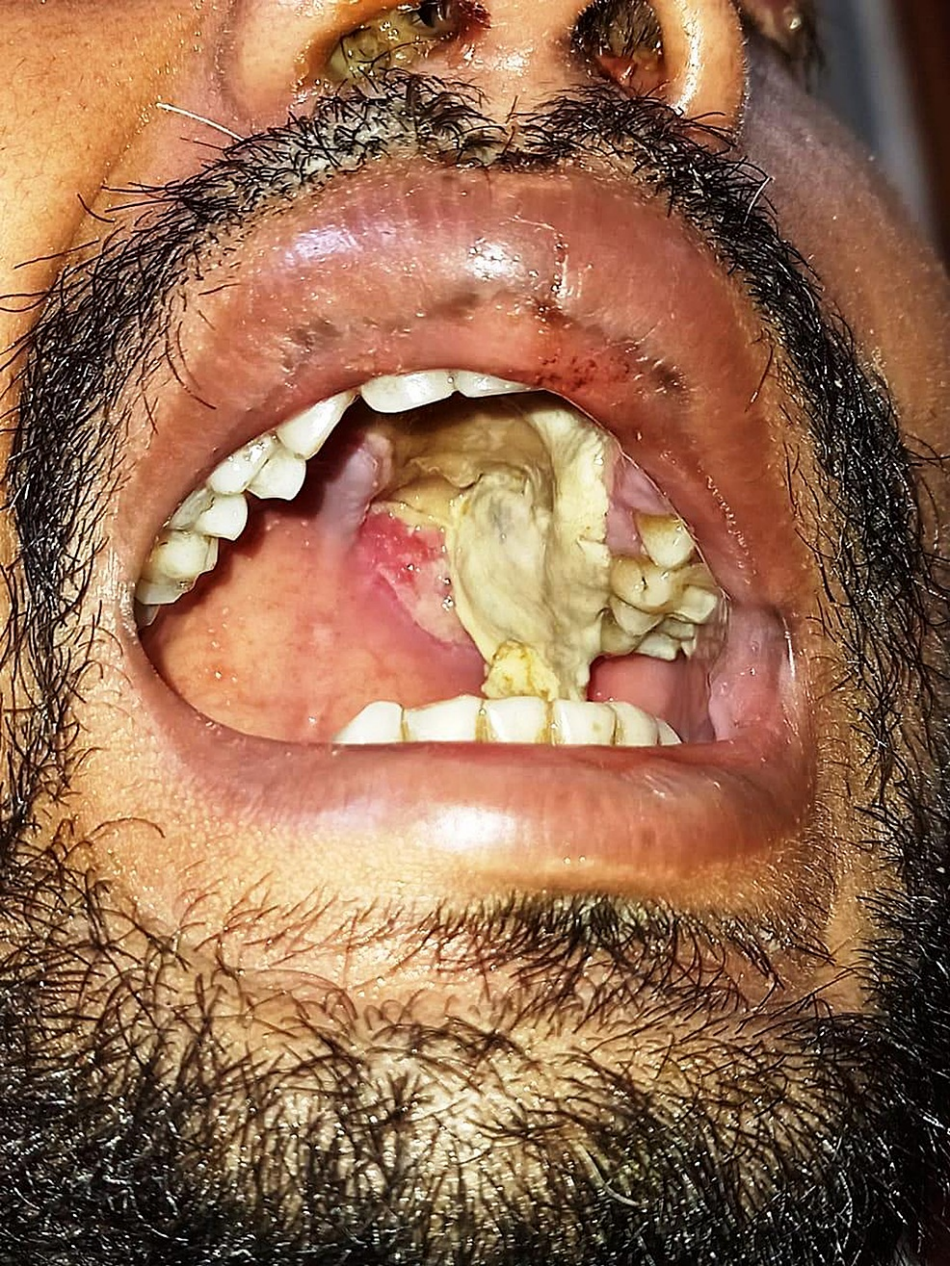
Mucormycosis involving the oral cavity

**Figure 2:**
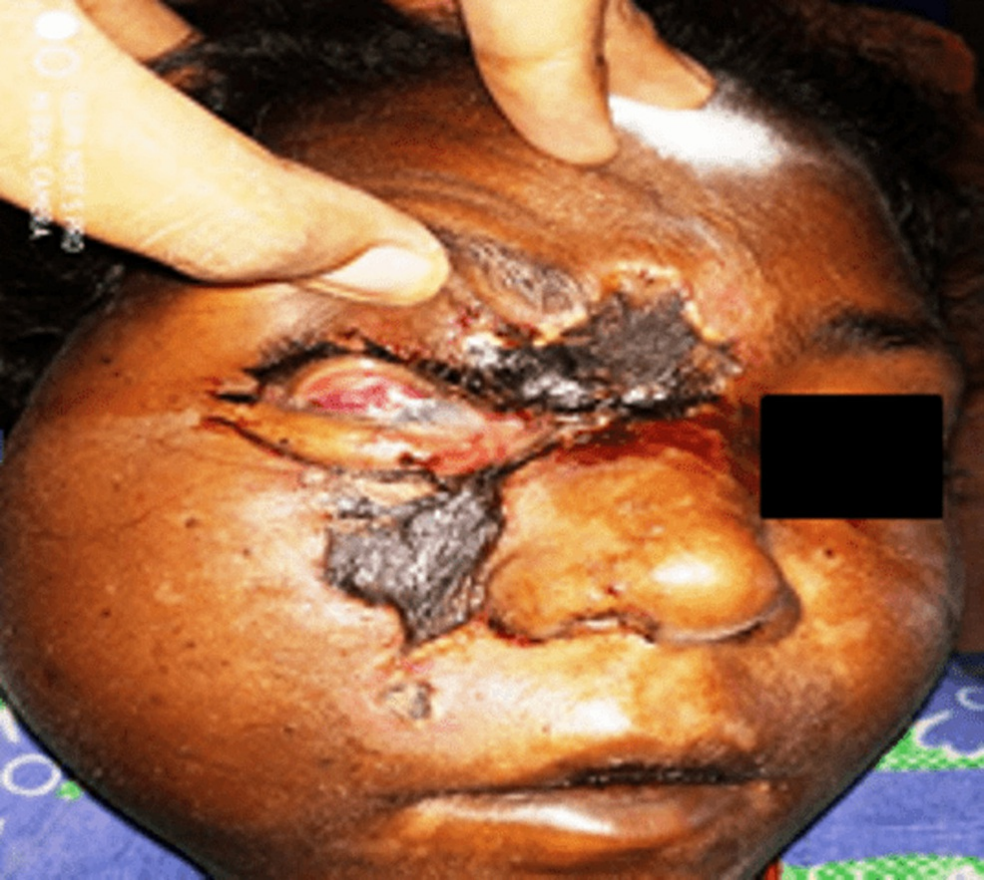
Mucormycosis involving orbit

Of 58 cases, 38 (65%) were males and 20 (35%) were females. Ages ranged from 34 to 77 years. SARS-CoV-2 was detected in 46 patients in RT-PCR with a CORADs score ranging from 4 to 6. Associated comorbidities were noted in 52 patients, with uncontrolled diabetes mellitus (46 patients; 88%) being the most common. The most common location of mucor infection was the nasal cavity and paranasal sinuses (43%) (Figure 1), followed by the orbital region (2%) (Figure 2), cerebral (10%) region and pulmonary area (8%). 

**Figure 3:**
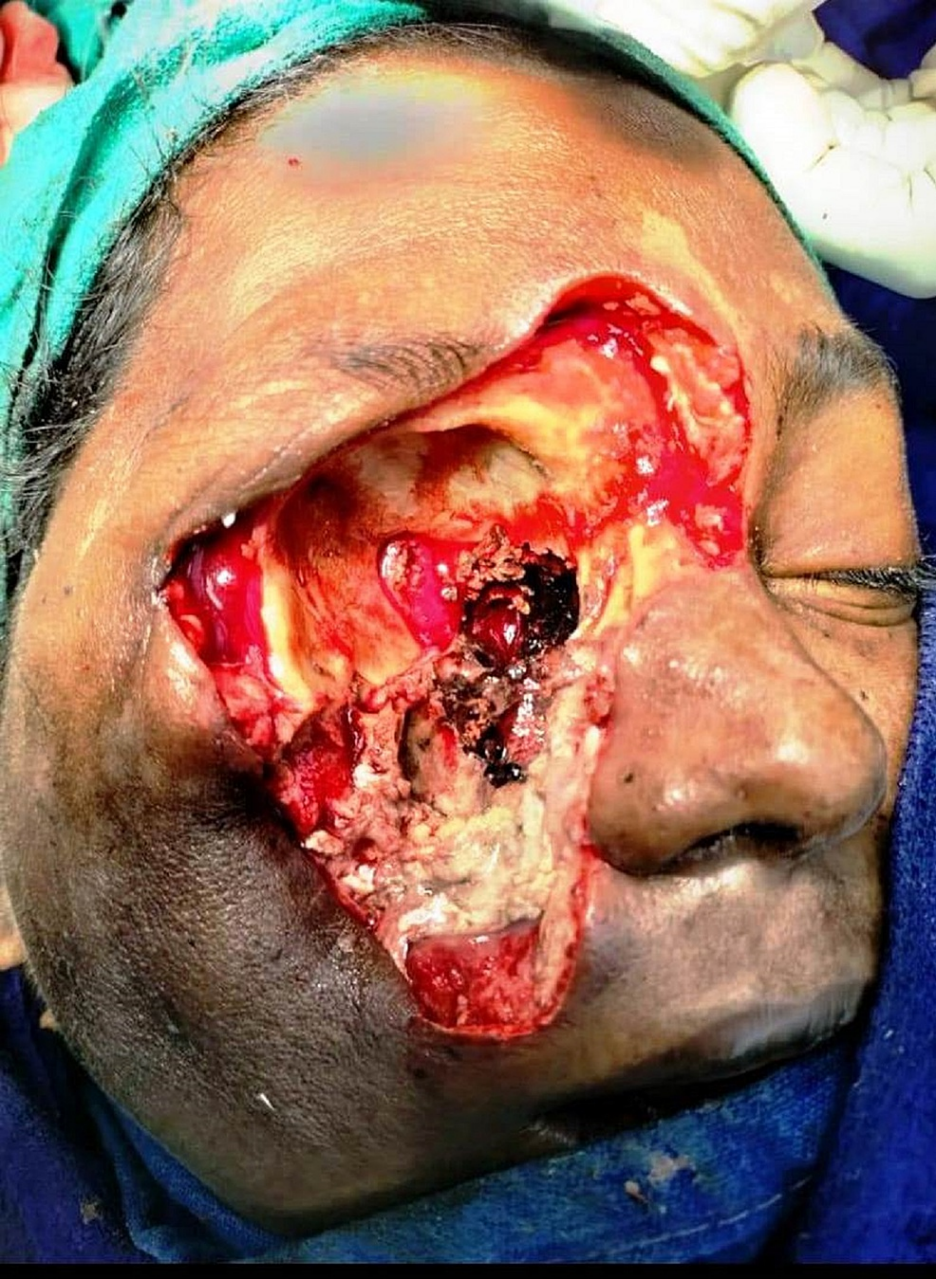
Post orbital exenteration

Among the paranasal sinuses, the maxillary sinus is commonly involved. Mixed fungal infections (Aspergillus sp. and Candida sp.) were noted in eight (14%) cases. Corticosteroid for the treatment of COVID-19 was administered in 49 patients. Tocilizumab in six patients and amphotericin B was administered in 59% of patients based on clinical findings alone and after histopathological evaluation. Ninety percent of them received amphotericin. Functional endoscopic sinus surgery (FESS) was done in 96% of cases. Among them 45% underwent extensive surgical debridement, 15% underwent orbital decompression and orbital exenteration (Figure 3) was done in 2% of cases (Table 1).

**Table 1:** Clinico-pathological parameters of 58 cases

Age (years)	34-77 years
Sex	
Male	38 (65%)
Female	20 (35%)
COVID-19 status	
RT PCR positive	30 (51.7%)
Negative	28 (48.3%)
Active	12 (20.6%)
Recovered	46 (79.4%)
Vaccination status	
2 doses completed	8 (13.7%)
Covishield	6
Covaxin	2
1 dose only	22 (37.9%)
Covishield	16
Covaxin	6
Not vaccinated	28 (48.2%)
Number of subjects who had reused disposable face masks	40 (68.9%)
CT SS for Covid pneumonia	
Normal	4 (6.8%)
Mild	9 (15.5%)
Moderate	22 (37.9%)
Severe	23 (39.6%)
Preexisting Airway and Sinus diseases.	31 (53%)
Treatment of COVID	
Steroid	48 (82.7%)
Tocilizumab	21 (36.2%)
Remdesvir	37 (63.7%)
Respiratory support	
O2 therapy	41 (70.6%)
Ventilatory support	24 (41.3%)
Location	
Paranasal Sinuses	42 (72.4%)
Intra orbital extension	22 (38%)
Intra cranial extension	3 (5.1%)
Lung	2 (3.4%)

Histopathology plays a major role as it not only distinguishes the presence of the fungus in the specimen from a culture contaminant but also is indispensible to define whether there is blood vessel invasion in the debrided tissue. It can furthermore reveal the presence of coinfections with other fungal organisms like Aspergillus sp. Mucorales genera produce typically non-pigmented, wide (5-20 µm), thin-walled, ribbon-like hyphae with no or few septations and a broad-angle branching, in contrast to Aspergillus species or other hyaline molds, which are typically 3-5 µm wide, septate and form acute-angle branching. Routine hematoxylin and eosin (H&E) stains show the presence of these hyphal forms in a necrotic background with a few invading the blood vessel walls and bony trabeculaes (Figure 3).

**Figure 4:**
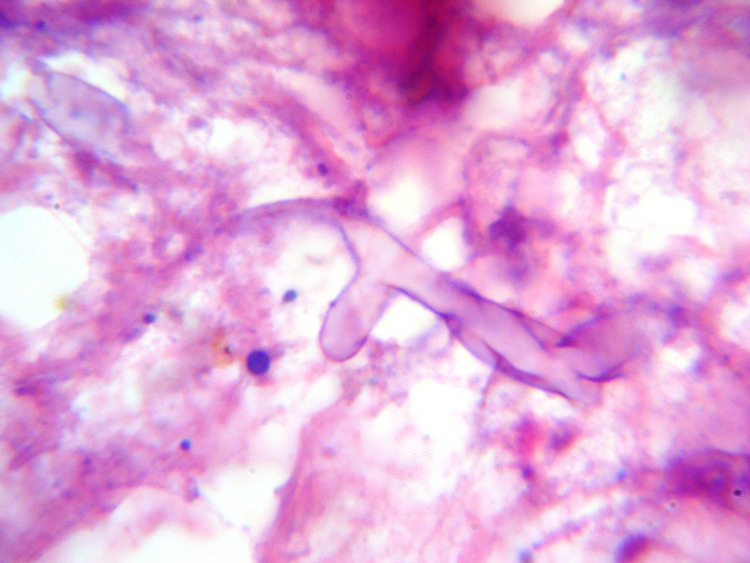
Mucor-Broad,aseptate fungal hyphae

Special stains that can help highlight the fungal wall include Grocott methenamine-silver (GMS) and periodic acid-Schiff (PAS), although PAS gives a better visualization of the surrounding tissue compared to GMS.[Figure4]

**Figure 5:**
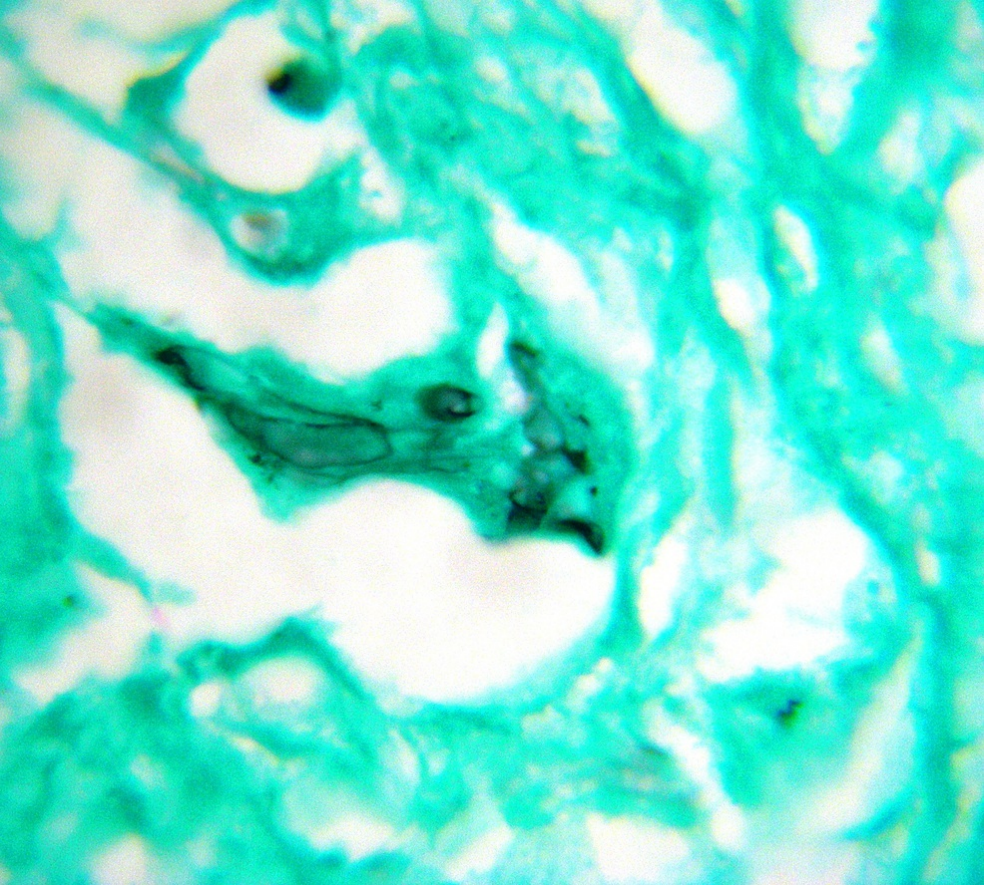
GMS stain highlighting the Mucormycosis

## Discussion

Mucorales are thermotolerant fungi, generally present in soil, decaying material, and immunocompetent people. The order Mucorales encompass 55 genera and 261 species, of which 38 are associated with infection in humans, the most common being Rhizopus. The primary route of entry is through nasal inhalation of aerosolized sporangiospores, which gets deposited in the paranasal sinuses. Removal of spores usually happens by sneezing or swallowing. The spores entering through breaches in mucous membrane are phagocytosed and destroyed by polymorphonuclear neutrophils. Hence patients with neutropenia have two times more risk of developing mucormycosis. Risk factors for infection include Immunocompromised states like diabetes mellitus with or without ketoacidosis, malignancies, organ transplants, immunosuppressive therapies, iron overload and AIDS. Other possible factors could be re-usage of disposable masks, quality of oxygen cylinders not par with medical compliance and poor sterilization of ventilators during the COVID-19 crisis.

Paranasal sinuses, central nervous system, lung, gastrointestinal system and skin are the usual sites of infection. The rapid progression and dissemination is attributed to the angioinvasive nature of the fungi. Clinical presentation depends on the site involved starting with nasal congestion and progressing on to frontal headache, facial numbness, ocular pain, blurry vision and diplopia indicating the invasion of orbital and cerebral areas [16-18]. Pulmonary mucormycosis manifests as cough, fever, hemoptysis and dyspnoea. It ranges from bilateral pneumonia to bronchitis and pulmonary embolism. From the lungs, the infection can spread to the mediastinum and heart [19]. The criteria for diagnosis of mucormycosis proposed by Smith and Kritchner [20] in 1950 are still considered as gold standard which includes (a) Black, necrotic turbinates appearing as dried, crusted blood, (b) Blood-tinged nasal discharge and facial pain on one side of the face, (c) Peri-orbital or peri-nasal swelling with discoloration and induration, (d) Ptosis of the eyelid, proptosis of the eyeball and complete ophthalmoplegia and, (e) Multiple cranial nerve palsies unrelated to documented lesions. Radiological evidence of invasion can be visualized as sinus opacification, bone erosion and obliteration of deep facial planes.

Histopathological evaluation remains the mainstay for diagnosis of mucormycosis. Mucormycosis appears as broad, aseptate or minimally septate ribbon-like hyphae ranging from 5 to 20 microns invading the blood vessels and bony trabeculae as shown.

Aspergillus sp. or other hyaline molds appears as septate hyphae with acute-angle branching ranging from 3 to 5 microns. On microscopy, these organisms are seen in areas of suppurative tissue necrosis. Host reaction includes dense inflammatory cell infiltrate predominantly composed of neutrophils, formation of giant cells and epitheloid granulomas. Special stains like Periodic acid-Schiff (PAS) or Grocott methenamine silver (GMS) can highlight the fungus as shown.

Direct microscopy using potassium hydroxide (KOH) wet mount enhances the visualization of the fungus. However fungal cultures help to identify the genus, species and antifungal susceptibility testing. Molecular methods like internal transcribed spacers (ITS) sequencing have emerged as useful tools for systematic analysis at the species level and within species as well. They are recommended as the first-line method for species identification in Mucorales [21].

Similar to other studies [22], our study showed a male preponderance of 38 (65%). The age group ranged between 34 to77 years with a median age of 44 years. Patel et al. [23] reported an increased risk of death in age >54 years in their study. Mixed fungal infections (Aspergillus sp. and Candida sp.) were noted in eight (14%) cases. In a study by White et al. [24] mixed fungal infections were noted in 26.7%, commonly aspergillosis (14.1%) and candidiasis (12.6%). In a recent review conducted by John et al. [25] that reported the findings of 41 mucormycosis cases in people with COVID-19, DM was reported in 93% of cases, while 88% were receiving corticosteroids. These findings are consistent with our findings where 88% of comorbid association was with DM (Table 2).

**Table 2:** Literature search of similar case series

Reference	Number Of cases	Age/ Range	Sex	Associated Comorbidities	Treatment	Location	Outcome
Sharma et al [8]	23	NR	M=15 F=8	DM(n=21)	Steroid (n=23)	Nasal sinus(n=23) Orbital(n=10) CNS(n=2)	Alive(n=21) LFU (n=2)
Moorthy et al [9]	17	39-73	M=15 F=2	DM(n=15)	Steroid (n=15)	Nasal sinus(n=17) Orbital(n=11) CNS(n=8) Bone(n=14)	Alive(n=7) Death(n=9) LFU (n=1)
satish et al [10]	11	30-74	M=NR F=NR	DM=majority Leukemia=1	Nil	Nasal sinus (majority) Orbital (majority)	Alive (n=4) Death(n=2) LFU(n=5)
Misra et al [11]	10	37-78	M=9 F=1	DM(n=8)	Steroid (n=6) Tocilizumab (n=1) Remdesvir (n=6)	Nasal sinus(n=10) Orbital(n=2) Bone(n=1)	Alive (n=5) Death(n=4) LFU(n=1)
Sarkar et al [12]	10	27-67	M=8 F=2	DM=10	Steroid (n=10) Remdesvir (n=5)	Nasal sinus(n=10) Orbital(n=10) CNS(n=1)	Unchanged (n=4) Alive (n=2) Death(n=4)
Sen et al [13]	6	46.2-74.9	M=6	DM=6	Steroid (n=5)	Nasal sinus(n=6) Orbital(n=6) CNS(n=1)	Alive & Improving (n=6)
Dallalzadeh et al [14]	2	36,48	M=1 F=1	DM=2	Steroid (n=2) Remdesvir (n=2)	Nasal sinus(n=2) Orbital(n=2) CNS(n=2)	Unchanged (n=1) Death (n=1)
Veisi et al [15]	2	40,54	M=1 F=1	DM=2	Steroid (n=2) Remdesvir (n=2)	Nasal sinus(n=2) Orbital(n=2) CNS(n=1)	Recovered (n=1) Death (n=1)
Present study	52	34-77	M-2 F-1	DM(n=46)	Steroid (n=49) Amphotericin n =52	Nasal & paranasal sinus(n=42) Orbital(n=22) Cerebrum(n=3) Lung(n=2)	Recovered(n=38) Death(n=7) Lost follow up(n=7)

Impaired bronchoalveolar macrophages, reduced CD4+ and CD8+ T cells, endothelial damage, destruction of pneumocytes and thrombosis associated with COVID-19 predisposes to secondary bacterial and opportunistic fungal infections. The ability of mucormycosis to survive in harsh conditions and evading detection by host immune mechanisms are attributed to the unique fungal cell wall. Spore coat protein homologs (CotH) of Mucorales aids in endothelial cell invasion, hematogenous dissemination and tissue necrosis. The pathogenic factors related to proteins and enzymes related to iron sequestration, upregulation of GRP78 cell receptor, support the survival and progression of mucormycosis inside the host. Enhanced expression of endothelial cell glucose-regulated protein 78 (GRP78) and CotH are associated with high glucose levels, low pH, free iron and ketones. Increased levels of IL-6 in COVID-19 increase the free iron levels by increasing ferritin levels. Diabetes mellitus is the highly prevalent immunosuppressed state in India. Diabetes mellitus with or without ketoacidosis during COVID-19 increases the risk of contracting mucormycosis. Also low pH due to acidosis and low oxygen saturation levels serve as a fertile media for the germination of sporangiospores. Moreover, steroids administered in the treatment of COVID-19 suppress the phagocytic activity of immune cells [26].

COVID-19 is associated with a significant incidence of a spectrum of secondary infections, both bacterial and fungal, probably due to immune dysregulation. Detailed analysis of clinicopathological features suggests the possibilities of immunosuppression (due to diabetes and use of corticosteroids in treatment of COVID-19) and COVID-19 (endothelial damage, cytokine storm) being the pathogenesis associated with the sudden surge of mucormycosis. Early diagnosis and treatment with the subsequent reduction of mortality and morbidity is the need of the hour. The use of therapeutic agents should be monitored to achieve a therapeutic effect at the lowest dose and shortest durations.

There are a few limitations to this study owing to considerable heterogeneity in reported cases. Few cases lack clinical details like duration of diabetes mellitus, HbA1c levels, ferritin levels, etc. Active and recovered COVID-19 cases and its relation to the onset of mucormycosis cannot be defined accurately due to lower sensitivity of confirmatory RT-PCR.

## Conclusions

Histopathology is the gold standard for diagnosis of mucormycosis. Complete clinic-pathological correlation will help us to efficiently treat the patients. Immunosuppressed individuals should be carefully monitored. Steroid and other immune-suppressive drug use should be used optimally. The uptrend in mucormycosis is mainly due to the triad of uncontrolled diabetes mellitus, rampant use of steroids and COVID-19 pathophysiology (cytokine storm and endothelial damage). All efforts should be made to control glucose levels, to appropriately use the steroids and finally early detection of mucormycosis both clinically and histopathologically.
